# The G119S *ace*‐1 mutation confers adaptive organophosphate resistance in a nontarget amphipod

**DOI:** 10.1111/eva.12888

**Published:** 2019-11-27

**Authors:** Kaley M. Major, Donald P. Weston, Michael J. Lydy, Kara E. Huff Hartz, Gary A. Wellborn, Austin R. Manny, Helen C. Poynton

**Affiliations:** ^1^ School for the Environment University of Massachusetts Boston Massachusetts; ^2^ Department of Integrative Biology University of California, Berkeley Berkeley California; ^3^ Center for Fisheries, Aquaculture and Aquatic Sciences Department of Zoology Southern Illinois University Carbondale Illinois; ^4^ Department of Biology University of Oklahoma Norman Oklahoma; ^5^ Department of Microbiology Harvard Medical School Boston Massachusetts; ^6^ Present address: Department of Environmental and Molecular Toxicology Oregon State University Corvallis Oregon

**Keywords:** evolution, *Hyalella azteca*, nontarget, organophosphate, pesticide resistance

## Abstract

Organophosphate (OP) and carbamate (CM) insecticides are widely used in the United States and share the same mode of toxic action. Both classes are frequently documented in aquatic ecosystems, sometimes at levels that exceed aquatic life benchmarks. We previously identified a population of the nontarget amphipod, *Hyalella azteca*, thriving in an agricultural creek with high sediment levels of the OP chlorpyrifos, suggesting the population may have acquired genetic resistance to the pesticide. In the present study, we surveyed 17 populations of *H. azteca* in California to screen for phenotypic resistance to chlorpyrifos as well as genetic signatures of resistance in the acetylcholinesterase (*ace*‐1) gene. We found no phenotypic chlorpyrifos resistance in populations from areas with little or no pesticide use. However, there was ~3‐ to 1,000‐fold resistance in *H. azteca* populations from agricultural and/or urban areas, with resistance levels in agriculture being far higher than urban areas due to greater ongoing use of OP and CM pesticides. In every case of resistance in *H. azteca*, we identified a glycine‐to‐serine amino acid substitution (G119S) that has been shown to confer OP and CM resistance in mosquitoes and has been associated with resistance in other insects. We found that the G119S mutation was always present in a heterozygous state*.* Further, we provide tentative evidence of an *ace*‐1 gene duplication in *H. azteca* that may play a role in chlorpyrifos resistance in some populations. The detection of a genetically based, adaptive OP and CM resistance in some of the same populations of *H. azteca* previously shown to harbor a genetically based adaptive pyrethroid resistance indicates that these nontarget amphipod populations have become resistant to many of the insecticides now in common use. The terrestrial application of pesticides has provided strong selective pressures to drive evolution in a nontarget, aquatic species.

## 1 INTRODUCTION

Organophosphate (OP) and carbamate (CM) pesticides have been widely used in the United States since the early 1970s when the U.S. Environmental Protection Agency (EPA) banned the organochlorine dichlorodiphenyltrichloroethane (DDT). Both OP and CM pesticides have the same mode of action, targeting acetylcholinesterase (AChE; EC 3.1.1.7). They elicit toxicity in insects by binding to AChE and preventing the breakdown of acetylcholine in the neuronal synapses, thus leading to over excitation of the nervous system, paralysis, and death (Aldridge, 1950). Human toxicity concerns associated with the OPs have led to the banning of some of these chemicals by the EPA (e.g., azinphos‐methyl, ethyl parathion) and severe restrictions placed on the use of others (methyl parathion). Among those restricted were the OPs chlorpyrifos and diazinon, for which all products intended for residential use were withdrawn from the marketplace in the early 2000s, though their agricultural uses continue. As a result of the restrictions on many OP products and general regulatory pressures to reduce their use, the annual use of OPs and CMs by professional pesticide applicators in California declined from a peak of nearly 8 million kg in 1995 to ~2 million kg in 2016 (CDPR, 1995, 1997, 2016) These quantities do not include residential home and garden use by nonprofessionals, which is not tracked by the state.

The widespread use of OPs and CMs poses a risk to aquatic ecosystems. Both pesticide classes are frequently detected in U.S. streams and rivers, sometimes at levels that exceed the established benchmarks for aquatic life and contribute significantly to urban stream impairment (Stone, Gilliom, & Ryberg, ). Historically, monitoring in California has found both urban and agricultural runoff to transport toxic concentrations of OPs into aquatic ecosystems (Bailey et al., 2000; Kuivila & Foe, 1995; Kuivila & Foe, 1995; Kuivila & Foe, 1995). In more recent years, OP concentrations in urban runoff have declined dramatically due to the cessation of residential use of diazinon and chlorpyrifos, and urban environmental concentrations of these compounds are now quite low (Weston, Holmes, & Lydy, 2009; Weston & Lydy, 2010). Toxicity due to OPs in California agricultural runoff is observed less frequently and is less widespread than it was in the 1990s, yet since that time, OPs from agricultural use have been implicated in acute toxicity to aquatic invertebrates including daphnids (*Ceriodaphnia dubia*), mayflies (*Procleon* sp), midges (*Chironomus dilutus*), and amphipods (*Hyalella azteca*) (Anderson et al., 2006). In addition, legacy OPs sequestered in stream sediments contribute to macroinvertebrate pesticide exposure as well (Rasmussen et al., 2015).

Pest insects can serve as models for understanding the effects of pesticides on aquatic organisms, especially given that insecticides do not discriminate between target and nontarget organisms in their mode of toxicity. In fact, the evolution of adaptive pesticide resistance caused by pesticide selective pressure is common among target pest insects (Feyereisen, Dermauw, & Van Leeuwen, 2015) and has been documented in some populations of the nontarget aquatic invertebrate *H. azteca* (Major, Weston, Lydy, Wellborn, & Poynton, 2018; Weston et al., 2013). In *H. azteca*, resistance to pyrethroid insecticides is both genetically based and predictable, with resistance occurring exclusively in waterways near land uses associated with pyrethroid applications (Major et al., 2018). In pyrethroid‐resistant *H. azteca* from urban or agricultural areas, resistance was explained by the presence of any of several mutations in the gene coding for the pyrethroid target site, the voltage‐gated sodium channel (*vgsc*) (Major et al., 2018; Weston et al., 2013). In one agricultural site with resident pyrethroid‐resistant *H. azteca* near Salinas, California (Chualar Creek), we found the OP chlorpyrifos at acutely toxic levels in the sediment at 13 times the 10‐day LC_50_ of sensitive laboratory *H. azteca* populations (Weston et al., 2013). Since pyrethroids and organophosphates have different modes of toxic action, the pyrethroid‐related mutations would be unlikely to confer resistance to chlorpyrifos. Thus, the persistence of the Chualar Creek *H. azteca* population in an environment with acutely toxic levels of chlorpyrifos suggests the population may have an additional adaptive mechanism providing OP resistance. However, the mechanism of that resistance has not yet been characterized.

Resistance to pesticides in some populations of *H. azteca* indicates a substantial pesticide presence in the environment capable of eliminating sensitive taxa. Pesticides acting as strong selective pressures may reduce genetic diversity via population bottlenecks and “genetic erosion” (Van Straalen & Timmermans, 2002). In pyrethroid‐resistant *H. azteca*, fitness costs including decreased thermal tolerance and greater sensitivity to other chemicals have been associated with resistance to pyrethroids (Heim et al., 2018). Further, increased bioaccumulation potential in pyrethroid‐resistant *H. azteca* and greater trophic transfer of pyrethroid residues to their fish predators have also been documented (Muggelberg et al., 2017). If some *H. azteca* have also evolved resistance to OP (and/or CM) pesticides, similar ecological and evolutionary costs could occur. Determining the extent of and mechanism behind the chlorpyrifos resistance in *H. azteca* as suggested by our Chualar Creek, observations must first be explored before we can fully comprehend the eco‐evolutionary impacts of pesticide use on these nontarget organisms.

In the present study, we use many of the populations from our previous work throughout California (Major et al., 2018) to screen *H. azteca* populations from agricultural and urban areas for chlorpyrifos resistance. Then, we investigate the mechanism of chlorpyrifos resistance in *H. azteca* by focusing on the gene that codes for the target site of OP and CM insecticides, acetylcholinesterase (*ace*‐1).

## 2 MATERIALS AND METHODS

### 2.1 Site selection

Wild *H. azteca* were obtained from 17 sites throughout California, mostly between October 2014 and November 2015 as recorded in Major et al. (2018), but with the collection of one population in January 2018 (Table S1). All sites are herein designated with a three‐letter code derived from the site name (e.g., Mosher Slough = MSH). The sites were a priori placed into one of three categories: (a) little or no OP or CM exposure expected, usually due to lack of development in the watershed (referred to as LowOCU sites); (b) extensive residential and commercial development in surrounding lands (Urban sites); and (c) intensive irrigated agriculture in surrounding lands (Agricultural sites). Some of the agricultural sites also had large population centers in the watershed, so would have some urban influence as well.

The rationale for these three groups can best be understood in light of current and historical OP and CM use in California. Table 1 compares their use from the present day (2016 most recent data available) to their use in 1995, representative of a period of high OP and CM use and prior to many regulatory restrictions since placed upon the OPs. In the 1990s, OP use was far greater than it is currently, and it was prevalent in both agricultural and urban environments. Much of the diazinon was used for residential purposes. Chlorpyrifos was primarily an agricultural pesticide, but since the total amount used annually in California was 1,500 metric tons, even the 18% applied in nonagricultural environments represented a substantial quantity. Malathion also had significant nonagricultural use. The withdrawal of nearly all diazinon‐ and chlorpyrifos‐containing products from residential use in the early 2000s dramatically changed the agricultural/urban use patterns for the OPs. Currently, only two OPs have significant nonagricultural use (malathion and naled), and since naled is used as a mosquito adulticide, much of the application is done in sparsely populated areas (e.g., rice fields, pasturelands). The CM pesticides are also used far less than had been the case historically. Yet unlike the OPs, very little of the compounds were ever used for nonagricultural purposes, and nearly, none are today.

**Table 1:** Annual use of organophosphate and carbamate pesticides in California, and the percentage of that use for nonagricultural purposes

Pesticide	1995 use (metric tons)	1995 use (% nonagric.)	2016 use (metric tons)	2016 use (% nonagric.)
Organophosphates				
Acephate	208	3	72	3
Azinphos‐methyl	184	<1	0	na^a^
Bensulide	31	13	133	<1
Chlorpyrifos	1536	18	409	<1
Diazinon	552	61^b^	22	<1
Dimethoate	271	<1	111	<1
Ethephon	446	<1	181	6
Malathion	364	18	161	12
Methamidophos	227	<1	0	na
Methidathion	146	0	<1	0
Naled	318	1	144	37
Phosmet	121	<1	13	<1
Profenofos	111	0	0	na
s,s,s‐tributyl phosphorotrithioate	393	0	3	0
Carbamates				
Aldicarb	161	0	0	na
Carbaryl	379	2^b^	100	<1
Carbofuran	110	0	0	na
Eptc	299	<1	116	0
Methomyl	366	<1	118	<1
Molinate	625	0	0	na
Pebulate	111	<1	0	na
Thiobencarb	254	0	317	0

Note

Values are compared from 1995 and the most recent year for which data are available (2016). Only compounds with greater than 100 metric tons use in either year are shown.

We define nonagricultural use to include landscape maintenance, structural pest control, protection of public health, regulatory pest control, treatment of rights‐of‐way, and application to golf courses. Agricultural use comprises use in the growing and processing of crops.

a The “na” indicates the percentage of nonagricultural use is not applicable since the total annual use is zero.

b Subsequent to their original publication, the California Department of Pesticide Regulation adjusted the 1995 use data to remove suspected erroneous entries. The 1995 use totals shown are the revised amounts (DPR 1997) rather than those originally published (DPR 1995). However, the percentages of nonagricultural use could only be calculated using the original data. For nearly all compounds listed, the adjustments were trivial and inconsequential to this analysis. However, for carbaryl and diazinon the adjustments resulted in reducing the annual use by nearly half. Thus, the percentage of nonagricultural use shown for these two compounds may not be accurate.

Thus, the four sampling sites comprising the “Urban” category would be expected to have had substantial OP exposure historically, but relatively little in approximately the past 15 years. The six sites in the “Agricultural” category would be expected to have both historical and ongoing exposure to OPs and/or CMs, though exposure concentrations likely have declined over the past couple decades. The seven sites in the “LowOCU” category would be expected to never have had significant exposure to either pesticide class. More detailed site descriptions can be found in Major et al. (2018), with the exception of the agricultural Ulatis Creek (ULC) site sampled in January 2018, which was not included in the previously mentioned publication. Further, the Russian River (RSN) site, characterized as “low use” in Major et al. (2018), is surrounded by agricultural land for wine grape production that does not rely on appreciable amounts of pyrethroids. This industry, however, does rely on OPs, and therefore, it was placed in the “Agricultural” category in the present study.

At each site, *H. azteca* were collected with a D‐frame net and transported with aeration to University of California Berkeley for toxicity testing with the OP chlorpyrifos within 1–3 days of collection (except 6 days for Chualar site). Either a single toxicity test or two concurrent independent toxicity tests were performed, depending on the number of individuals available. Random individuals from the testing group were also set aside in ethanol for later genetic analysis. When a sufficient number of individuals for toxicity testing were not available at a site, *H. azteca* were preserved in ethanol for genetic analysis only. Testing data were also obtained from a laboratory population of *H. azteca* maintained at the University of California Berkeley since 2003, and of the same U.S. Lab Strain (Major, Soucek, Giordano, Wetzel, & Soto‐Adames, 2013) as in Weston et al. (2013) and Major et al. (2018).

### 2.2 Analysis of chlorpyrifos in sediment and water

For most sites, with the exception of Outlet Creek (OTL), surficial sediment (0–2 cm) was collected and sent to Southern Illinois University for chlorpyrifos analysis (Table S1). Water samples from chlorpyrifos toxicity tests (see below) were also sent for analysis. Chlorpyrifos extractions followed the methods previously detailed elsewhere for sediment (Weston, Chen, & Lydy, 2015; You, Weston, & Lydy, 2008) and water (Wang, Weston, & Lydy, 2009). Once extracted, all samples were analyzed on an Agilent 6850 gas chromatograph 5975 XL mass spectrometer (GC‐MS; Agilent Technologies) with methane negative‐ion chemical ionization and selected‐ion monitoring. Organic carbon content for sediments was determined by drying the sediments, removing the inorganic carbon by acid vapor treatment, and analyzing samples on a CE‐440 elemental analyzer from Exeter Analytical (Chelmsford, MA).

### 2.3 Chlorpyrifos toxicity testing

Field‐collected and laboratory‐cultured *H. azteca* were challenged with the OP chlorpyrifos in 96‐hr water‐only exposures. Organisms were size‐fractionated prior to testing, and preference was given to juveniles that were able to pass through a 600‐µm screen, but were retained on a 500‐µm screen. For populations in which sufficient juveniles within that size range were unavailable, larger animals retained on a 1,000‐µm screen, but passing through a 2,000‐µm screen were used. Rostrum‐to‐telson body length measurements were recorded for approximately 30 individuals per population (Table S2).

Chlorpyrifos sensitivity was assessed using the 96‐hr water‐only acute toxicity methods as described in Major et al. (2018). Briefly, three replicate beakers, each containing 80 ml of media, 10 *H. azteca* and a 1‐cm^2^ piece of nylon mesh substrate were prepared for each treatment concentration. Because of difficulty with gender identification in *H. azteca* before euthanizing animals, gender was not scored before the toxicity tests were initiated. Base media consisted of Milli‐Q deionized water reconstituted with salts and bromide (Borgmann, 1996; Smith, Lazorchak, Herrin, Brewer‐Swartz, & Thoney, 1997). Media was used to create serial dilutions of chlorpyrifos in an acetone carrier, using 2× concentration steps. Solvent controls always contained <40 µl/L acetone. Testing conditions were 23°C (except ULC at 19°C) with a 16:8 light:dark photocycle. Each beaker received 1 ml of yeast, cerophyll, trout food on the second day of the test, and a 4‐hr feeding period allowed before media was replaced with fresh treatment solution (with the exception of beakers in the ULC test which were unfed). At test termination after 4 days, survivors (those individuals showing movement) were counted and LC_50_ values derived by the trimmed Spearman–Karber method, using CETIS (Tidepool Scientific Software). Survivorship over 90% is often used as a threshold for test acceptability when testing standard laboratory‐cultured *H. azteca*, and this threshold was met in the majority of our tests. However, given that the testing was done using wild‐collected individuals from diverse habitats throughout California, and the animals transported back to the laboratory, some relaxation of the survivorship threshold is reasonable. We provide control survivorship data for every test (Table S2) and include all tests exceeding 70% survival.

Testing of some populations yielded a small number of individuals that appeared healthy and exhibited normal behavior at concentrations far above those that had caused mortality to the majority of the test population. These were termed “survivors” and were set aside in ethanol for later analysis to determine the genetic basis for their insensitivity to chlorpyrifos toxicity. They are designated herein with an “S” suffix after the site name of the population (e.g., Mosher Slough survivors = MSH_S).

For each toxicity test, water was analyzed for chlorpyrifos from one concentration in the mid‐point of the testing range. Each sample for water analysis included media from test initiation composited with the mid‐test water change. Actual concentrations ranged from 69% to 120% of nominal concentrations. Reported LC_50_s were adjusted based on the actual, measured concentration of chlorpyrifos in that specific test, rather than nominal concentration.

### 2.4 Genomic DNA extraction

Genomic DNA (gDNA) was extracted from individual *H. azteca* stored in ethanol. Preference was given to extracting males whenever possible to reduce the likelihood that offspring from a gravid female would contribute to the gDNA profile of an individual. When it was not possible to use only males, care was taken to prevent eggs from being transferred into the extractions. All extractions followed the Qiagen DNeasy® Blood & Tissue Kit (Qiagen) protocol with slight modifications documented in Major et al. (2018), including a steel‐bead maceration step after initial buffer and proteinase K addition and an overnight incubation at 56°C. After extraction, gDNA was measured for purity (260/280 ratio) and nucleic acid concentration with a spectrophotometer (NanoDrop 2000; Thermo Scientific).

### 2.5 COI genotyping

*Hyalella azteca* is known to be a species complex; thus, a 670‐bp segment of the mitochondrial cytochrome *c* oxidase I (COI) gene was genotyped for a subset of individuals (5–10) from laboratory and wild populations to determine species identity within the complex. For most animals in the present study, species identity had previously been determined and can be found in Major et al. (2018). Some animals, such as the chlorpyrifos toxicity test survivors as well as the ULC wild population collected in January 2018, had not been previously characterized. For those individuals, COI genotyping methods followed those in Major et al. (2018). Briefly, the COI segment was PCR‐amplified using primer pairs IV, V, or VI (Table S3). GoTaq Green Master Mix (Promega Corporation, Madison, WI) with standard protocols was used with 40 µl reaction volumes. Cycling conditions were 5 min at 94°C; 40 cycles of 30 s at 94°C, 30 s at 52°C, and 45 s at 72°C; and 5 min at 72°C. PCR products were gel‐purified and sequenced with primer VII (Table S3) using an ABI 3730 automated sequencer.

### 2.6 *H. azteca* species determination using two gene segments

COI sequences were primarily used for species determination in the present study, but in cases where COI sequences were not available for all individuals, a segment of a nuclear marker, the *vgsc*, was used to infer species affiliation as established and validated in Major et al. (2018). For most of the animals from wild populations in the present study, *vgsc* sequences came directly from the Major et al. (2018) study, and when they did not (ULC population and select chlorpyrifos toxicity test survivors), the same methods were employed to obtain *vgsc* sequences. Briefly, a segment of the *vgsc* (578 bp in laboratory individuals) was PCR‐amplified in 50 µl reaction volumes, consisting of 25 µl Phusion Hot Spot II High Fidelity Green Taq Polymerase Master Mix (Thermo Fisher Scientific), 17.5 µl nuclease‐free water, 2.5 µl of 10 µM primer pair VIII (Table S3), and 5 µl of individual *H. azteca* gDNA. Thermocycler settings were 98°C for 30 s; 35 cycles of 98°C for 10 s, 64.2°C for 30 s, and 72°C for 30 s; 72°C for 10 min. PCR products were verified on a gel and cleaned with the QIAquick PCR Purification Kit (Qiagen) with a 40 µl elution volume, and 200–300 ng was sent to the Massachusetts General Hospital DNA Core (Cambridge, MA) for sequencing with one of three internal primers (primers IX through XI, in that order; Table S3). Manual sequence cleaning and heterozygote base pair calls were made based on a secondary peak cutoff value of 30% amplitude of the primary peak or higher. Vgsc loci M918 and L925 were scored for the wild ULC individuals to document potential pyrethroid resistance alleles in this wild population not previously included in Major et al. (2018).

A 326‐bp *vgsc* alignment of 175 *H. azteca* individuals was created including animals from wild population surveys (those from Major et al. (2018) plus the January 2018 ULC population) and chlorpyrifos toxicity test survivors using MUSCLE in MEGA v 7.0 (Kumar, Stecher, & Tamura, 2016). After alignment, PhyML (Guindon et al., 2010; http://www.atgc-montpellier.fr/phyml/) was used to generate a maximum likelihood (ML) tree. The substitution model (HKY85 + G, gamma shape parameter = 0.264) was chosen automatically based on Akaike information criterion (AIC = 2,620). Branch supports of greater than 90% (10,000 bootstrap replicates) were retained and displayed on branches (Figure S1) of an unrooted cladogram. Both COI and *vgsc* data were available for some individuals in the present study. In those cases, species determinations based on analysis of COI segments were overlaid onto the *vgsc* cladogram (Figure S1). Based on these distinctions, the highly supported branches of the *vgsc* trees were used to infer species affiliation for those individuals that were not sequenced at COI.

A minority of individuals from the present study, with available, but insufficient *vgsc* sequence quality or length (28), were excluded from the *vgsc* ML analysis, and thus, no species determinations could be directly inferred for those individuals. However, for sites at which only one species was identified, we assumed the remaining individuals at that site belonged to the same species group. For sites with more than one species present, COI and/or *vgsc* evidence was used to make species‐level distinctions for all individuals.

### 2.7 Target site gene cloning and resistance allele discrimination

To identify possible mutations associated with chlorpyrifos resistance in wild *H. azteca,* a segment of the acetylcholinesterase (*ace*‐1) gene was PCR‐amplified and cloned for several individuals from select resistant and nonresistant populations. A single *ace* gene (with homology to *ace*‐1 in insects), composed of three exons including two coding regions and a 5’ UTR, was identified in the U.S. Lab Strain *H. azteca* genome (Poynton et al., 2018). Predicted protein sequences of *H. azteca* AChE were aligned with the *Torpedo californica* AChE mature enzyme amino acid sequence to identify the regions in the *H. azteca* protein that corresponded to resistance mutations documented in other insects, as reviewed by Fournier (2005). Given that all mutations associated with or conferring resistance in other insects were located on the largest exon of the *H. azteca ace*‐1, a 906‐bp segment of that exon was targeted for PCR amplification and cloning. To capture the potential variation among *ace‐*1 sequences in wild and laboratory‐cultured *H. azteca*, one individual from a laboratory population (UCB), two to three from each of select wild populations (AMR, MSH, and CLG), and three to five chlorpyrifos toxicity test survivors from resistant populations (MSH_S and CLG_S) were selected for segment amplification and cloning. Sex was not recorded, but animals were more likely to be males than females based on our preferential selection of males for genotyping. Primer pair I (Table S3) was designed using the *H. azteca* genome *ace*‐1 as a template and Primer3 v. 0.4.0 (Untergasser et al., 2012). PCRs (20 µl) consisted of 10 µl Brilliant III Ultra‐Fast SYBR^®^ Green QPCR Master Mix with Low ROX (Agilent Technologies, Santa Clara, CA), 1 µl of 10 µM primer pair I (Table 3.1), 4 µl nuclease‐free water, and 5 µl gDNA. Thermocycler settings were 95°C for 5 min; 35 cycles of 95°C for 30 s, 58.5°C for 30 s, and 68°C for 45 s; 68°C for 15 min. Fresh PCR products were confirmed on an agarose gel and then cloned using the pCR®4‐TOPO® TA Cloning® Kit with One Shot® TOP10 Chemically Competent *E. coli* (Invitrogen, Carlsbad, CA). Five clones per individual were picked and screened for the desired amplicon and then grown in LB broth and purified using the QIAprep Spin Miniprep Kit (Qiagen). Primers T3 and T7 (Table S3) were used to sequence clones at the MGH DNA Core Facility (Cambridge, MA).

Cloned sequences were cleaned and aligned using CLC Main Workbench v 7.9.1 (https://www.qiagenbioinformatics.com/) and translated to amino acid sequences using MEGA v.7.0.20 (Kumar et al., 2016). Translated sequences were again aligned to mature *T. californica* AChE enzyme amino acid sequences to screen for amino acid substitutions potentially associated with chlorpyrifos resistance as reviewed by Fournier (2005).

### 2.8 *Ace‐*1 genotyping assay

An *ace*‐1, direct genotyping assay was created based on the identification of a candidate resistance allele at amino acid position G119 (*T. californica* numbering) in select wild populations of cloned *H. azteca* (MSH, MSH_S, CLG, and CLG_S). As with amplicon cloning, primer pair I (Table S3) was used to PCR amplify the *ace*‐1 segment using a high fidelity polymerase. PCRs had the same reagents, volumes, and settings as those listed for amplification of the *vgsc* segment (above), with the exception of the use of primer pair I instead of primer pair VIII (Table S3). After bands were confirmed on an agarose gel, they were cleaned with the QIAquick PCR Purification Kit (Qiagen) with a 40 µl elution volume. Between 200 and 300 ng of each cleaned PCR product was sent to the Massachusetts General Hospital DNA Core (Cambridge, MA) for sequencing with one of two internal primers. Internal sequencing primers were designed based on highly conserved *ace*‐1 regions across the individuals using Primer3 v. 0.4.0 (Untergasser et al., 2012). Sequencing with Primer II produced a 575 bp sequencing read while a secondary primer (used in rare cases of primary primer failure; Primer III) produced a 352 bp read (Table S3).

Because an individual's *ace‐*1 alleles were sequenced concurrently (as in the *vgsc* genotyping assay in Major et al. (2018)), resulting sequences were manually examined and cleaned using IUPAC ambiguity codes. All sequences were cleaned, aligned, and trimmed using CLC Workbench v. 7.9.1 (https://www.qiagenbioinformatics.com/), and the resulting G119 genotype was scored by manual visualization of the sequence. Heterozygotes were indicated by a double peak at a single locus. When a secondary peak was 10% of the height of the primary peak or less at a locus, it was discarded as noise and the sequence was repeated with an alternative primer until a clean sequence was obtained. When secondary peaks were 10% of the height of the primary peak or greater, the individual was scored as a heterozygote at that locus. The *ace*‐1 genotyping assay was verified by comparing genotyping assay results to the genotype profiles of the 16 individuals from which *ace*‐1 amplicons were cloned. Comparison showed that *ace*‐1 assay genotypes were the same as those recorded from cloning for all individuals. In the present study, between 10 and 20 individuals from each of the survey populations of *H. azteca* were assayed for *ace*‐1 genotype, including chlorpyrifos test survivors.

## 3 RESULTS

### 3.1 Chlorpyrifos in sediment

**Figure 1:**
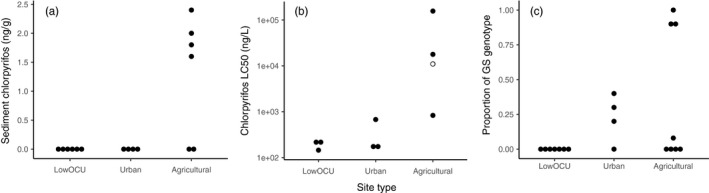
Panel (a) Dotplot of chlorpyrifos sediment concentrations at all sampling sites. When undetected, concentration shown as zero. Panel (b) Chlorpyrifos water‐only 96‐hr LC50 from all wild populations tested. Black dots represent measured values, using medians for those populations tested multiple times. The hollow dot is a “greater than” LC_50_ value for which the highest test concentration yielded <50% mortality. Panel (c) Proportion of *H. azteca* with an acetylcholinesterase (*ace*‐1) GS genotype. Only populations with five or more individuals shown

Chlorpyrifos was detected at concentrations of ~2 ng/g in the sediments from four of the six Agricultural sites (MSH, CHL, CLG, and WHW). Chlorpyrifos was not detected at any of the LowOCU or Urban sites (Figure 1a; Table S1).

### 3.2 Chlorpyrifos Sensitivity

The median chlorpyrifos 96‐hr LC_50_ for the UCB laboratory‐cultured population was 154 ng/L (Table 2). Populations from all three LowOCU sites tested were comparably sensitive to chlorpyrifos with median 96‐hr LC_50_s of 145–235 ng/L, and in most cases, with 95% confidence intervals overlapping those of the laboratory population (Table S2).

**Table 2:** Proportion of individuals with a given acetylcholinesterase (*ace*‐1) genotype (wild‐type wt) or resistant (res)) in a survey of wild *H. azteca* from sites in California and chlorpyrifos toxicity test survivors

Population	Code	Median population chlorpyrifos 96‐hr LC_50_ (ng/L)	Species	Sample size (*n*)	Proportion of individuals with a given genotype at G119	
					GG (wt)	GS (res)
Laboratory animals						
University of California Berkeley Laboratory	UCB	154	C	10	1.00	‐
Low organophosphate and carbamate use (LowOCU) expected						
Bassey Spring Creek	BSC	na	E	10	1.00	‐
Little Shasta River	LSH	200	Ps17	10	1.00	‐
Outlet Creek	OTL	na	B	10	1.00	‐
Burcham Creek	BCM	na	Ps28	4^c^	1.00^c^	‐
Owens River	OWN	145	Ps28	10	1.00	‐
South Fork Kern River	KRN	na	D	10	1.00	‐
Mojave River	MJV	235	D	10	1.00	‐
Urban Sites						
American River	AMR	161	B	20	1.00	‐
Medea Creek	MED	676	D	10	0.80	0.20
Buena Vista Creek	BVS	na	C	10	0.70	0.30
Escondido Creek	ESC	188	C	10	0.60	0.40
Agricultural Sites (with or without urban influence as well)						
Russian River	RSN	na	B	5	1.00	‐
			F	5	1.00	‐
Ulatis Creek	ULC	17,800	D	10	0.10	0.90
Mosher Slough	MSH	831	B	8	1.00	‐
			D	12	0.92	0.08
Chualar Creek	CHL	>11,000^a^	D	10	‐	1.00
Calleguas Creek	CLG	156,000	D	10	0.10	0.90
Whitewater Creek	WHW	na	C	10	1.00	‐
Chlorpyrifos test survivors^b^						
Mosher Slough, 1,870 ng/L	MSH_S	831	B	2^c^	1.00^c^	‐
			D	11	‐	1.00
Calleguas Creek, 272,000 ng/L	CLG_S	156,000	D	10	‐	1.00
Medea Creek, 6,650 ng/L	MED_S	676	D	10	0.10	0.90
Escondido Creek, 1,640 ng/L	ESC_S	188	C	10	‐	1.00

Note

“na” is not assessed. “‐” indicates a value of zero. Values are median LC_50_s for populations tested multiple times.

a This value is an average of two toxicity tests for which LC_50_s could not be obtained due to mortality <50% at the highest concentration, one with the highest concentration of 1,520 ng/L and the other at 20,500 ng/L. The actual LC_50_ for these populations could not be determined.

b Values following population name indicate the concentration of chlorpyrifos survived by these individuals that appeared unimpaired at the end of the 96‐hr test.

c Allele frequencies from populations with fewer than five individuals should be regarded with caution.

Of the three Urban sites, only one exhibited even a modest degree of chlorpyrifos resistance. The MED population had an elevated chlorpyrifos LC_50_, approximately fourfold greater than that of the UCB laboratory population and threefold greater than the least chlorpyrifos‐sensitive of the LocOCU populations (MJV). The LC_50_s of all the other Urban sites fell within a range (161–188 ng/L), similar to populations with no prior chlorpyrifos exposure.

All four of the Agricultural populations challenged with chlorpyrifos exhibited elevated LC_50_s, often dramatically so. The least‐elevated LC_50_s of the Agricultural sites were MSH, where the chlorpyrifos tolerance was 4–6 times that of sensitive populations (Table 2, Figure 1b). At all other Agricultural sites, the wild *H. azteca* were 47 to 1,000 times less sensitive to chlorpyrifos than the laboratory or sensitive wild populations. The LC_50_ of the ULC population was 17,800 ng/L and that of the CLG population was 156,000 ng/L. It was not possible to calculate a true LC_50_ at CHL because the highest test concentration used of 20,500 ng/L was insufficient to produce 50% mortality.

### 3.3 *H. azteca* species determination and pyrethroid resistance mutations

Analysis of COI sequences supported seven species groups previously reported by Major et al. (2018), designated as species B, C, D, E, F, Ps 17, and Ps 28 (Table 2). Pairwise COI sequence divergence between species groups ranged between 10% and 23% (Major et al., 2018).

Overall, species identity did not explain the differences in chlorpyrifos sensitivity generally observed between LowOCU, Urban, and Agricultural populations. *H. azteca* from LowOCU sites were members of species B, D, E, Ps17, and Ps28, those from Urban sites were species B, C, and D, and those from Agricultural sites were species B, C, D, and F. The *ace*‐1‐genotyped survivors of chlorpyrifos toxicity tests were members of the same species group(s) as the overall population tested from their respective sites (Table 2). Only two sites yielded organisms with more than a single species group: RSN and MSH. In the initial MSH collection, ratios of the B and D species groups were relatively balanced (12 D and 8 B; Table 2), while a higher proportion of species D remained among chlorpyrifos survivors (11 D and 2 B).

In using the *vgsc* segment as a tool for species determination, we were also able to use these sequences to genotype for pyrethroid resistance alleles for the one wild population not previously included in Major et al. (2018). All ULC individuals were species D and had at least one pyrethroid resistance allele (either M918L, L925I, or L925V; Table S4). Frequencies of mutations were 0.15, 0.80, and 0.05 for M918L, L925I, and L925V, respectively.

### 3.4 Variation in *ace‐1*

Cloning of the *ace*‐1 alleles for sixteen *H. azteca* individuals yielded 20 different alleles (based on 876‐bp alignments; Figure S2 through Figure S3), with most individuals showing evidence of more than two *ace*‐1 alleles (Table 3). The documented allele diversity was unlikely to be due to polymerase error, as base pair changes at a given site were screened and only considered true changes if other *H. azteca* clones varied similarly at the same site. If a base pair change was observed in a single allele but was never documented in any other allele from other populations, it was discarded as polymerase error (with the exception of Allele 3 in UCB—see below). Thus, our approach to delineate alleles was conservative. In addition, only five clones were sequenced from each individual, also likely leading to a conservative estimate of total alleles per individual.

**Table 3:** Number of alleles by acetylcholinesterase (*ace*‐1) G119S genotype documented in cloned *H. azteca* individuals

Population	Individual	No. of *ace*‐1 alleles	No. of G119wt alleles	No. of G119S alleles
Laboratory population				
UCB	1	3	3	0
Low organophosphate and carbamate use (LowOCU) expected				
AMR	1	1	1	0
	2	1	1	0
Agricultural sites				
MSH	1	3	3	0
	2	2	1	1
			
CLG	1	3	2	1
	2	5	2	3
	3	2	1	1
Chlorpyrifos test survivors				
MSH_S	1	3	2	1
	2	3	1	2
	3	2	1	1
			
CLG_S	1	4	2	2
	2	3	2	1
	3	3	1	2
	4	3	2	1
	5	2	1	1

Note

See Table 2 for population abbreviations.

All alleles had high homology to one another in both nucleotide (97.3% and greater) and amino acid sequences (98.3% and greater), providing evidence that alleles were all *ace*‐1, as opposed to *ace*‐2, which has not been found in the *H. azteca* genome (Poynton et al., 2018), but is common in other arthropods. The single laboratory individual for which *ace*‐1 amplicons were cloned had three *ace*‐1 alleles, one of which included a premature stop codon (Allele 3, Figure S4). It is possible that the base pair substitution leading to the stop codon was a polymerase error given that it was not found in any other clones, but the allele also had distinct motifs that corresponded to the *ace*‐1 consensus sequence in the *H. azteca* genome, making it a unique allele even in the absence of a true premature stop codon. Taken together, the evidence that single individuals harbor three or more alleles suggests that recent gene duplication of the *ace‐*1 gene has occurred in the majority of the studied populations, including in the U.S. Lab Strain. Alternatively, polyploidy as a result of variation in genome sizes among wild *H. azteca* (Vergilino, Dionne, Nozais, Dufresne, & Belzile, 2012) could be an alternate reason for evidence of multiple *ace*‐1 alleles in some wild populations.

Only three amino acid residues associated with resistance in insects (as reviewed by Fournier (2005)) were identified in the cloned *H. azteca* in the present study at positions 119, 128, and 129 (relative to *T. californica* nomenclature). Two were fixed in both resistant and nonresistant *H. azteca* populations (E128 and V129), thus not contributing to the LC_50_ variation observed among populations. The G119S mutation (always produced by codon AGC) was documented in a total of seven different cloned alleles, and only from populations that had elevated LC_50_s (MSH and CLG), including survivors from high concentrations in the chlorpyrifos toxicity tests (MSH_S and CLG_S). Surviving individuals always had at least one G119S allele, and G119S was always in a heterozygous state (never in all alleles for a given individual). The *ace*‐1 G119S mutation was originally documented in CM‐resistant mosquitoes (Weill et al., 2003), but it has also been shown to also confer resistance to OPs (Essandoh, Yawson, & Weetman, 2013; Liebman et al., 2015).

Five additional amino acid substitutions were documented in the cloned *ace‐*1 amplicons of *H. azteca*, each occurring in four alleles or fewer. M236L and S256N mutations (numbering relative to *T. californica*) were identified in alleles from populations with elevated chlorpyrifos LC_50_s and/or survivors (Figure S3). However, these substitutions have not been identified as contributors to OP or CM resistance, and their association with resistance requires further study. The remaining amino acid substitutions, L346M, I379L, and K386R, were present in nonresistant and resistant populations (Figure S4), with no evidence of resistance conferred in other insects. Because of prominence of the G119S mutation in all resistant animals, and the uncertainty of the role of any additional substitutions only found in a few individuals, we focused on the G119S for our genotyping assay.

### 3.5 Proportion of OP resistance genotypes in *H. azteca*

A total of 237 *H. azteca* individuals from wild and laboratory populations and survivors of chlorpyrifos toxicity tests were successfully genotyped at *ace*‐1 (see Supporting Information “ace.txt” for sequence alignment). The *ace*‐1 genotyping assay was effective for scoring the G119 locus for at least 10 individuals per site including members from all seven species groups identified in the present study (B, C, D, E, F, Ps17, and Ps28) with a single exception. At Burcham Creek (BCM; Ps 28), only four of 10 individuals were successfully genotyped (Table 2). Although it was not possible to determine the total number of *ace*‐1 alleles from the genotyping assay data alone, the G119S mutation always occurred in a heterozygous state, with no SS genotype detected in any individual. Further, the G119S mutation was always the result of the same base pair substitution (GGC to AGC). It was identified in both species C and D organisms, indicating a single origin followed by introgressive hybridization or at least two independent origins of the allele in *H. azteca*.

**Figure 2:**
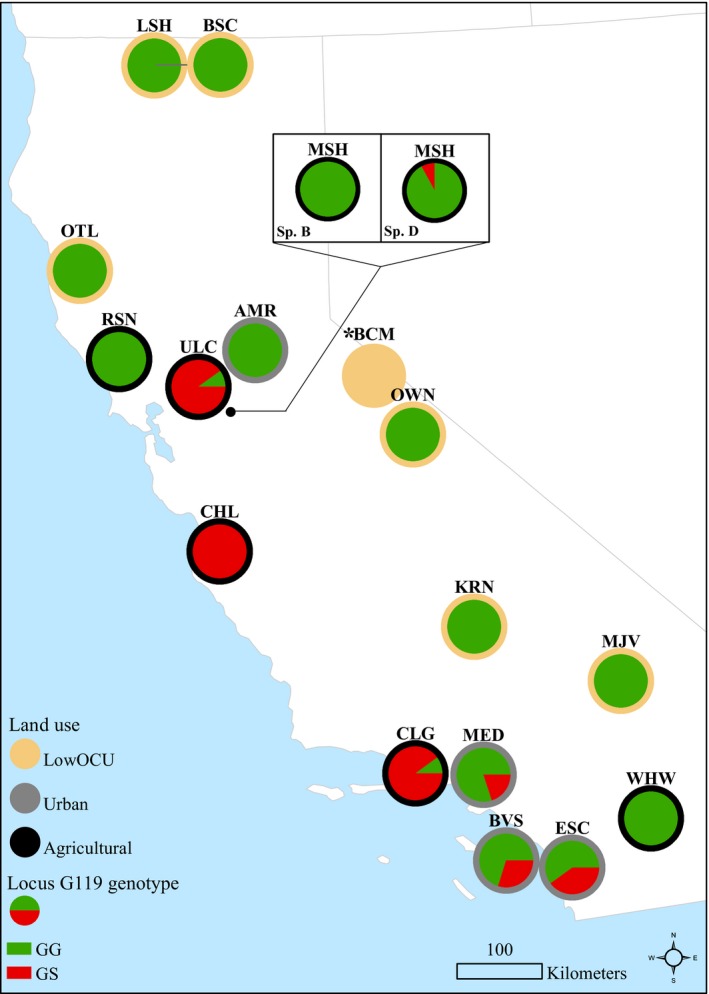
Map of the acetylcholinesterase (*ace*‐1) genotype (GG or GS) proportions for each *H. azteca* population. LowOCU sites are outlined in tan, Urban sites are outlined in gray, and Agricultural sites are outlined in black (see Table 2 for site abbreviations). For BCM, designated by an asterisk (*), fewer than five individuals were genotyped at *ace*‐1, and proportions are not displayed. When two or more species were identified at a site and they differed from one another in G119S genotype composition, proportions for both species are shown

**Figure 3:**
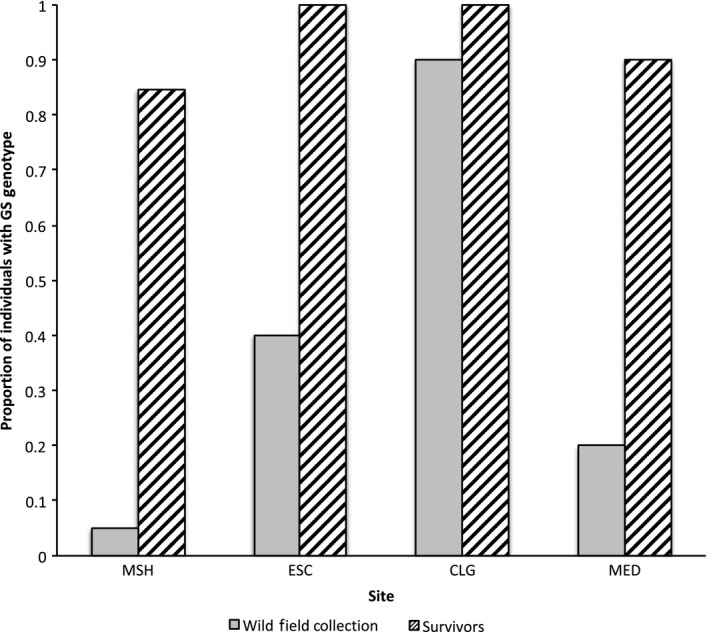
Proportion of individuals listed by site with acetylcholinesterase (*ace*‐1) GS genotype before (initial wild collection) and after (survivors) a 96‐hr chlorpyrifos water‐only challenge. Proportions are not separated by species group (see Table 2 for site abbreviations), although only a single species (either C or D) was identified at ESC, CLG, and MED sites. For MSH, presented proportions represent the pooled sample of species B and D

Populations that harbored individuals with a GS genotype were found in Urban and Agricultural sites only, and never in populations where little or no prior OP and CM exposure was expected, including all 64 individuals tested from the LowOCU sites or in 10 individuals from the UCB laboratory population (Table 2, Figure 1c). At least one individual with a GS genotype was detected in most populations from Urban (three of four) and Agricultural (four of six) sites (Figure 2). Urban populations generally had a lower proportion of individuals with the GS genotype than did Agricultural populations (Table 2). The three Agricultural sites with the highest LC_50_s, (CHL, CLG, and ULC; all species D), also had the highest proportion of individuals with GS genotypes among their individuals collected from the field (0.90 or higher; Table 2). The proportion of individuals with a GS genotype was always higher among chlorpyrifos toxicity challenge survivors than in the control population for a given Urban or Agricultural site (Figure 3). In most instances, the difference in resistant genotype frequencies between the population overall and the survivors was dramatic (e.g., resistant genotype found in 8% of the species D from Mosher Slough overall, but in 100% of the Mosher species D survivors; resistant genotype found in 20% of the Medea Creek individuals overall, but in 90% of the Medea survivors).

## 4 DISCUSSION

In the present study, we screened wild populations of *H. azteca* for resistance to the OP chlorpyrifos. Chlorpyrifos resistance in *H. azteca* is geographically widespread in California. Five different populations, spanning a geographic distance of over 500 km within the state, displayed a phenotypic chlorpyrifos resistance between threefold and 1,000‐fold greater than sensitive laboratory or wild populations, and we found increased tolerance largely in areas of agricultural land use. We showed that a single nucleotide polymorphism producing an amino acid substitution (G119S) in the target site for chlorpyrifos was associated with every instance of measured resistance in *H. azteca* and appeared in two species groups. The allele was never detected in sensitive laboratory or wild (LowOCU) populations that were likely to have received little or no prior exposure to OP or CM pesticides. Furthermore, within a given population, those individuals capable of surviving high concentrations of OP in a controlled laboratory exposure always had a disproportionately higher frequency of the resistance allele than in the general population from which they came. We provided evidence that the resistance observed in *H. azteca* is related to an *ace*‐1 GS genotype. Given that individuals from most populations tested had three or more *ace*‐1 alleles, an *ace*‐1 gene duplication or polyploidy may play a role in resistance, although the genomic architecture of resistant *H. azteca* remains uninvestigated. The genetically based, adaptive chlorpyrifos resistance in the nontarget *H. azteca* revealed through this study raises the possibility of potential fitness costs to populations affected by OP and CM runoff.

### 4.1 An* ace‐1* mutation is associated with every case of chlorpyrifos resistance in *H. azteca*

We found a glycine‐to‐serine amino acid substitution (G119S) in Urban and Agricultural sites that was most prevalent in *H. azteca* populations with an elevated resistance to chlorpyrifos, and was in nearly all individuals that were able to survive chlorpyrifos exposures up to 10 times the recorded LC_50_ for a population, or in organisms from Urban and Agricultural sites. The proportion of individuals with a GS genotype was always higher in survivors from the chlorpyrifos toxicity test than in the general wild population, providing strong evidence that this mutation is associated with the development of resistance to chlorpyrifos. While it is possible that sensitivity in *H. azteca* could vary as a function of species group as sometimes reported (Leung, Witt, Norwood, & Dixon, 2016; Soucek, Mount, Dickinson, Hockett, & McEwen, 2015), the wide range of chlorpyrifos sensitivity among *H. azteca* in the present study was best explained by the presence of a GS genotype and not by species group. Further, the starting proportion of individuals with the GS genotype in wild populations was generally related to the level of resistance in the population. Urban populations with no or moderate chlorpyrifos resistance had a minority of individuals in the wild populations with the GS genotype, while the most resistant Agricultural populations with extreme chlorpyrifos resistance (47‐ to 1000‐fold greater LC_50_s) had had high proportions of the GS genotype (≥0.90). Thus, we have demonstrated through multiple lines of evidence that the G119S mutation in *H. azteca* was associated with phenotypic resistance to chlorpyrifos.

While we measured chlorpyrifos sensitivity in populations containing both sexes, we preferentially genotyped males in order to eliminate the possibility of screening mate DNA through inclusion of offspring in the DNA preps. In other aquatic crustaceans, pesticide resistance is sex‐linked (Carmona‐Antonanzas et al., 2017). However, despite our preference for males, we genotyped females from many sites, including from populations in all categories (LowOCU, Urban, Agricultural, and Survivors) and in all species groups. The G119S mutation did not appear to be associated with sex; thus, the role that sex plays in OP resistance in *H. azteca*, if any, cannot be elucidated by the present study.

The same G119S mutation was originally documented in the *ace*‐1 of mosquitoes (*Culex pipiens* and *Anopheles gambiae*). In vitro assays using recombinant wild‐type and mutant enzymes showed that insensitivity to propoxur (a CM) in *C. pipiens* was explained by this single amino acid substitution (Weill et al., 2003). The glycine‐to‐serine substitution occurs in the oxyanion hole near the catalytic triad, reducing access of the insecticide to the target site (Weill, Malcolm, et al., 2004). Although the first example of resistance through G119S‐conferred target site insensitivity was identified using a CM, it has been widely established that this mutation confers both OP and CM resistance across multiple species of mosquitoes all over the world (Essandoh et al., 2013; Liebman et al., 2015). The G119S mutation has also been associated with chlorpyrifos resistance in another insect, the brown planthopper (*Nilaparvata lugens*). Chlorpyrifos treatment of a field‐collected population for nine generations produced a resistant strain (253‐fold greater LC_50_) with the G119S mutation (Zhang, Yang, Li, Liu, & Liu, 2017). Taken together, the detection of G119S in Agricultural and Urban populations of *H. azteca* (but not in populations with no prior OP or CM exposure expected), as well as its characterization as a resistance allele in multiple mosquito species and the brown planthopper establish evidence of a genetic, adaptive basis for chlorpyrifos resistance in *H. azteca*.

In *H. azteca* from the present study, we only identified the G119S mutation in a heterozygous state. Among the 237 individuals genotyped at *ace*‐1, we found no individuals harboring an SS genotype despite a large number with the GS genotype (78). Our finding suggests that the SS genotype has a strong fitness cost, potentially indicating the SS genotype in *H. azteca* is nonviable. In mosquitoes, the GS genotype shows overdominance (heterozygote advantage) in which individuals with the G119S allele are more fit in the presence of an OP or CM, but they are less fit in the absence of those contaminants. In the absence of an insecticide, the G119S allele reduces AChE activity by 60% in *C. pipiens* (Bourguet et al., 1997). Other studies with mosquitoes have documented fitness costs associated with the G119S allele, including reproductive costs (Berticat et al., 2008) and developmental and physiological costs (Bourguet, Guillemnaud, Chevillon, & Raymond, 2004). The homozygous form of the G119S allele has been connected to a higher mortality rate for pupae in *A. gambiae* (Djogbenou, Noel, & Agnew, 2010). If, as these multiple studies with other arthropods have shown, the SS genotypes in *H. azteca* suffer a high fitness cost that prevents their development into adulthood, then the past OP and/or CM exposure has imposed a significant cost on the reproductive capacity of the wild *H. azteca* populations.

The identification of the G119S mutation in both species C and D animals is evidence of a single emergence in one species group followed by introgressive hybridization or at least two independent origins of the mutation in *H. azteca*. In mosquitoes, the G119S mutation has also been independently and repeatedly selected multiple times as a result of OP and CM selective pressure (Weill, Berthomieu, et al., 2004; Weill et al., 2003), although some evidence of introgression as a mechanism for the spread of G119S across species groups exists (Djogbenou et al., 2008). However, introgression is only a plausible mechanism for transferring resistance alleles when two species can mate to produce viable offspring. Forced crosses in our laboratories do not produce fertile offspring (M. Lydy, personal communication), making adaptive introgression highly unlikely. Our results further suggest that interbreeding in wild populations containing two separate clades (Major et al., 2018; Weston et al., 2013) does not occur, supporting independent origins of resistance in *H. azteca* across species groups (Major et al., 2018). The existence of sensitive, wild‐type species C (laboratory) and species D (Mojave River) combined with the potential fitness cost associated with the G119S allele provides support that selection for G119S has occurred by OP and CM exposure rather than based on shared common ancestry given the high (14%) COI divergence between species C and D animals (Major et al., 2018).

### 4.2 Evidence for an* ace‐1* duplication in *H. azteca*

In the small subset of *H. azteca* cloned at a*ce‐*1 in the present study, we found evidence of between three and five different alleles for some individuals, with Agricultural group individuals (Calleguas Creek and Mosher Slough) sometimes harboring multiple versions of the G119S allele (two to three). The high homology (>97% nucleotide sequence identity) among all alleles (G119S or wild‐type) suggests that these alleles come from a duplication of the *ace*‐1 gene rather than an *ace*‐2 gene, consistent with the failure to detect an *ace*‐2 gene in the *H. azteca* genome (Poynton et al., 2018).

A precedent for *ace*‐1 duplication exists for some OP‐ and CM‐resistant organisms. In mosquitoes (*A. gambiae* and *C. pipiens*), multiple independent *ace*‐1 gene duplications have produced a new allele (*ace* ^D^) with a copy of the susceptible allele and a copy of the resistance allele in tandem on the same chromosome (Djogbenou et al., 2008; Djogbenou, Labbe, Chandre, Pasteur, & Weill, 2009; Labbe et al., 2007; Lenormand, Guillemaud, Bourguet, & Raymond, 1998). A recent study of the genomic architecture of the *ace*‐1 duplication showed that duplications are associated with all resistance alleles in *A. gambiae,* indicating that *ace*‐1 duplication is an important mechanism for resistance in some mosquitoes (Assogba et al., 2016). In resistant mosquitoes, the ace^D^ allele allows for fixed heterozygosis and has been shown to resorb many of the fitness cost associated with the G119S mutation (Assogba et al., 2015). In crustaceans, a duplication of the *ace*‐1 gene was found in the salmon louse (*Lepeophtheirus salmonis*), which has developed resistance to OPs through a different point mutation in one of the *ace*‐1 copies (Kaur, Helgesen, Helgesen, Bakke, & Horsberg, 2015). This provides the first example of a recent *ace*‐1 duplication within this taxonomic group (Kaur, Bakke, Bakke, Nilsen, & Horsberg, 2015), although more work is needed to determine whether there is a relationship between the duplication and OP selective pressure.

It is possible, however, that the detection of multiple *ace*‐1 alleles is instead explained by polyploidy rather than an *ace*‐1 duplication event. Within the *H. azteca* species complex, genome size can vary by species group and evidence of polyploidy in some North American groups of *H. azteca* exists (Vergilino et al., 2012). However, the genome size variation and potential of polyploidy for groups in the present study remain uninvestigated, and future work will be needed to determine the genomic architecture associated with the multiple *ace*‐1 alleles documented herein.

In the present study, we only cloned and sequenced a limited number of individuals. Given that the *ace*‐1 genotyping assay did not allow for a discrete characterization of all alleles for individuals, we cannot fully define the role of multiple *ace*‐1 alleles as a resistance mechanism in *H. azteca.* However, the presence of multiple sensitive and resistant *ace*‐1 alleles in some cloned Mosher Slough and Calleguas Creek chlorpyrifos test survivors suggests that an *ace* ^D^ allele may exist in some *H. azteca*, and that copy number variation may play a role in resistance. In the chlorpyrifos survivors, nearly all *H. azteca* had a GS genotype, but some individuals did not appear to have multiple alleles suggesting that a multiple *ace*‐1 alleles may not be a requirement for resistance. Interestingly, evidence for the existence of multiple alleles was limited to species C (a single individual from the UCB laboratory population) and species D (Calleguas Creek and Mosher Slough) animals, the same species groups in which the G119S allele was identified, although more individuals need to be sequenced at *ace*‐1 to confirm this observation. Unfortunately, no species C *ace*‐1 amplicons from resistant organisms were cloned to determine whether an *ace*‐1 duplication was present in those populations. Thus, more work is needed to characterize the role of the potential gene duplication in *ace*‐1 or polyploidy in resistant populations and individuals.

It is worth mentioning that while the high variability of resistance phenotypes (threefold to 1000‐fold) among populations harboring the GS allele may be explained by the proportion of individuals with a GS genotype or the presence of multiple GS alleles in individuals or the population, other mechanisms may play a role in resistance as well. These alternate mechanisms could include the upregulation of detoxification genes (acclimation) or other adaptive mechanisms (e.g., mutations in detoxification genes, decreased penetration of OPs) that were not assessed in our populations. We screened the majority of the largest of the three *ace*‐1 exons in *H. azteca*, and it is possible that additional mutations in the other coding region or the 5’UTR are also contributing to resistance phenotypes. Although the portion that we genotyped covered most of the known resistance mutations in other insects, it is possible that a previously undescribed mechanism for resistance exists in *H. azteca*, and more work is needed to determine the full mechanism(s) of resistance.

### 4.3 Chlorpyrifos resistance in *H. azteca* is predictable based on exposure

Our results were in good agreement with our a priori established site groupings. The LowOCU sites where little OP and CM exposure was expected based on surrounding land uses contained *H. azteca* populations with no phenotypic resistance to chlorpyrifos, and these populations lacked the G119S mutation.

At Urban sites, we expected high OP exposure prior to the early 2000s, but minimal exposure for approximately the past 15 years, since the regulatory restrictions on diazinon and chlorpyrifos use. The *H. azteca* Urban populations generally showed no resistance to chlorpyrifos, and only a modest level (threefold) of resistance in one of four sites. While the G119S mutation was found in Urban locations, it was in only a minority of the individuals (0%–40% depending on site). It is possible that the low frequency of mutation is a relic of past use of diazinon and chlorpyrifos, maintained even in the absence of present high selective pressures through mechanisms such as a gene duplication. It is also possible the persistence of the mutation is indicative of ongoing low‐level exposure. In California, chlorpyrifos remains detectable in urban runoff, but well below acutely toxic levels for *H. azteca* (Weston et al., 2009; Weston & Lydy, 2010, 2012). As noted earlier, other OPs, such as naled or malathion, could still be in use in California urban environments. In a recent U.S.‐wide survey, the OP dichlorvos and the CM carbaryl were frequently detected in urban streams from 2002 to 2011, with at least occasional exceedances of aquatic life benchmarks (Stone et al., ).

Chlorpyrifos remains one of the more widely used insecticides in California agriculture and is by far the most heavily used of the OPs. Therefore, as expected we found the greatest phenotypic resistance in the Agricultural sites (all four sites, up to 1000‐fold resistance). The mutant genotype was found in four of six Agricultural sites and was present in 90%–100% of the individuals at three of them. The data suggest a strong, ongoing selective pressure for the G119S mutation in many agriculture‐influenced areas, driven by chlorpyrifos and/or other compounds within the OP and CM classes.

In a study of mutations conferring pyrethroid pesticide resistance in *H. azteca*, pyrethroid residues were consistently found in all sites where use of the compounds had been anticipated, and were often present at concentrations above acutely toxic thresholds for wild‐type individuals, thus providing supportive chemical evidence of a selective pressure (Major et al., 2018). However, in the present study, chlorpyrifos was only found in four of the six Agricultural sites, and only at very low concentrations. Given a reported organic carbon‐normalized chlorpyrifos sediment LC_50_ of 1.77 µg/g organic carbon (Amweg & Weston, ), the measured concentrations in the present study equate to only approximately 5% of the reported LC_50_, failing to demonstrate a selective pressure (though not ruling out selection operating through other than acutely lethal mechanisms). The success of the pyrethroid sediment data in supporting the genetic and toxicological findings and the absence of a comparable relationship with chlorpyrifos are likely due to differences in the physical/chemical properties of the compounds. Pyrethroids are extremely hydrophobic and adsorb tightly to sediments. Log K_oc_ values for the pyrethroids are typically in the range of 5 to 6 (Laskowski, 2002). Chlorpyrifos is less hydrophobic, with a K_oc_ of 3.9 (Solomon et al., 2014). We did not even pursue sediment analyses of the other OPs and CMs of interest in the present study because their log K_oc_s are even lower, typically 2–3 (MacKay, Shiu, & Ma, 1997). In addition, pyrethroids persist in sediments longer than chlorpyrifos, particularly under aerobic conditions (Budd, O'Geen, Goh, Bondarenko, & Gan, 2011). Thus, sediments are inherently a much better integrator of pyrethroid exposure history at a given site than they are for chlorpyrifos or any of the other OPs and CMs of interest. In fact, data exist that indicate our Agricultural sites have been exposed to far more chlorpyrifos than the low concentrations of the present study suggest. The Chualar Creek sediment contained 2 ng/g chlorpyrifos when sampled in 2014 for the present study, but it contained 248 ng/g when sampled in 2010 (13 times the organic carbon‐adjusted LC_50_ for wild‐type *H. azteca*; Weston et al., 2013). The Mosher Slough sediment contained 1.8 ng/g when sampled in 2014 for the present study, but contained 25 ng/g in 2010 (approximately half the LC_50_; M. J. Lydy, unpublished data).

### 4.4 Resistance in* H. azteca* has ecological and evolutionary implications

Although the potential fitness costs associated with OP‐ and CM‐resistant *H. azteca* have not been explored, the G119S allele has been associated with reduced AChE functionality (Bourguet et al., 1997) and reduced fitness in mosquitoes (Assogba et al., 2015). However, the existence of *ace* ^D^ allele in mosquitoes is capable of absorbing much of the G119S cost of resistance measured as *A. gambiae* larval mortality and development time, mating competition, and female fecundity and fertility (Assogba et al., 2015). This suggests that fitness costs associated with the G119S allele may also be ameliorated in fixed heterozygote *H. azteca* with duplicated *ace*‐1 genes. However, other population‐ or community‐level costs may be associated with the G119S allele, especially when considering that seven populations from the present study harbored both pyrethroid resistance alleles (M918L, L925I, and/or L925V; data shown in Major et al. (2018) and in Table S4) and the OP and CM resistance allele (G119S). For example, pyrethroid‐resistant *H. azteca* can act as a vector for pyrethroid bioaccumulation in fish (Muggelberg et al., 2017). Therefore, chlorpyrifos bioaccumulation of chlorpyrifos and its metabolites in fish predators may also be applicable for fish feeding on pesticide‐resistant *H. azteca*, resulting in the potential for increased fish bioaccumulation not only of pyrethroids, but of OPs and/or CMs. Further, it is possible that selective sweeps for chlorpyrifos resistance have contributed to the reduced fitness already observed in some pyrethroid‐resistant *H. azteca* (Heim et al., 2018). More research is warranted to determine the full extent of population and community‐level effects of OP resistance.

The ecological implications of resistance are not limited to *H. azteca* populations or the animals directly connected to this amphipod in the food web. Chlorpyrifos from agricultural runoff has been documented at levels that were acutely toxic to a variety of other ecologically important aquatic invertebrates including daphnids (Anderson et al., 2003), chironomids, and mayflies (Anderson et al., 2006). Given that such toxicity could act as the driver behind the development of pesticide resistance, it is possible that other species are undergoing similar OP and CM adaptive processes as those documented in *H. azteca*.

## 5 CONCLUSION

Agricultural and urban OP and CM use has been the driver behind the adaptive, genetically based chlorpyrifos resistance observed in numerous populations of the nontarget aquatic amphipod, *H. azteca*. Resistant populations all share the same glycine‐to‐serine amino acid substitution at position 119 of *ace*‐1 also found to be one of the primary mutations involved in mosquito OP and CM resistance. Sensitive laboratory and wild populations with no history of pesticide exposure lacked the G119S mutation. This mutation has developed independently at least twice in two species groups within *H. azteca* species complex. Further, although our study design possessed limited ability to discriminate alleles at *ace*‐1, we found that an *ace*‐1 duplication (or polyploidy) may be common in some members of the *H. azteca* species complex and may even aid in resistance to OPs and CMs. The full extent of population and ecosystem‐level impacts for chlorpyrifos‐resistant *H. azteca* requires further study, but the existence of genetically based chlorpyrifos resistance throughout California indicates that OP and/or CM pesticides used in agricultural and urban settings have left a genetic signature of evolutionary pressure, the full effects of which have not yet been elucidated. Most of the current widely used insecticides fall within four classes: OPs, CMs, pyrethroids, and neonicotinoids. All the populations demonstrated here to have OP and CM resistance also have been shown to be resistant to pyrethroids (Major et al., 2018; Weston et al., 2013). Their sensitivity to the neonicotinoids has not yet been investigated, but they clearly have acquired resistance to many of the insecticides now in common use. This finding provides clear evidence that pesticide use throughout the state and the subsequent movement of those residues into aquatic systems have had a profound effect on evolution in *H. azteca*.

## Conflict of interest

None declared.

## Data Availability

Data used in these analyses and primer sequences used for sequencing are provided in the Supplemental Information.
