# Supplementary material for: The G119S ace‐1 mutation confers adaptive organophosphate resistance in a nontarget amphipod
Source: Evol Appl. 2019 Nov 27;13(4):620–35. doi: 10.1111/eva.12888 (PMC7086107; doi:10.1111/eva.12888)
Supplement: Supplementary file 1 [file EVA-13-620-s001.docx]

**Supporting Information**

|  | Site Name | Watershed | Latitude, Longitude | Land Use*^a^* | Sampling Date | OC (%) | Chlorpyrifos (ng/g) |
| --- | --- | --- | --- | --- | --- | --- | --- |
| **Low organophosphate and carbamate use (LowOCU) expected** | | | | | |  |  |
|  | Bassey Spring Creek (BSC) | Klamath River to Pacific Ocean | 41.7172 , -122.3471 | F, R | June 2015 | 2.42 | nd |
|  | Little Shasta River (LSH) | Klamath River to Pacific Ocean | 41.7114 , -122.3833 | F, R, C*^b^* | June 2015 | 6.82 | nd |
|  | Outlet Creek (OTL) | Eel River to Pacific Ocean | 39.5319, -123.4050 | F | June 2015 | --^c^ | --^c^ |
|  | Burcham Creek (BCM) | Walker River to endorheic basin | 38.3840 , -119.4295 | F, R | July 2015 | 16.50 | nd |
|  | Owens River (OWN) | Owens River to endorheic basin | 37.7499, -118.9379 | F | July 2015 | 2.29 | nd |
|  | South Fork Kern River (KRN) | Kern River to endorheic basin | 35.6908, -118.2348 | F, R, C*^b^* | May 2015 | 4.41 | nd |
|  | Mojave River (MJV) | Mojave River to endorheic basin | 35.0380 , -116.3814 | D | May 2015 | 0.64 | nd |
| **Urban Sites** | | | | | |  |  |
|  | American River (AMR) | Sacramento River to San Francisco Bay | 38.5658 , -121.3838 | U | Nov. 2014 | 3.26 | nd |
|  |  |  |  |  |  |  |  |
|  | Medea Creek (MED) | Malibu Creek to Pacific Ocean | 34.1401, -118.7600 | U | June 2015 | 3.90 | nd |
|  |  |  |  |  |  |  |  |
|  | Buena Vista Creek (BVS) | Buena Vista Creek to Pacific Ocean | 33.1814, -117.3218 | U | June 2015 | 1.68 | nd |
|  |  |  |  |  |  |  |  |
|  | Escondido Creek (ESC) | Escondido Creek to Pacific Ocean | 33.0986, -117.1306 | U | June 2015 | 0.77 | nd |
| **Agricultural Sites (with or without urban influence)** | | | | | |  |  |
|  | Russian River (RSN) | Russian River to Pacific Ocean | 38.5664 , -122.8515 | U, C | Aug. 2015 | 1.48 | nd |
|  | Ulatis Creek (ULC) | Sacramento River to San Francisco Bay | 38.3068, -121.7939 | U, C, R | Jan. 2018 | 1.76 | nd |
|  | Mosher Slough (MSH) | San Joaquin River to San Francisco Bay | 38.0325, -121.3654 | U, C | Oct. 2014 | 1.70 | 1.8 |
|  | Chualar Creek (CHL) | Salinas River to Pacific Ocean | 36.5583, -121.5296 | C | Oct. 2014 | 2.06 | 2.0 |
|  | Calleguas Creek (CLG) | Calleguas Creek to Pacific Ocean | 34.1644, -119.0612 | U, C | July 2015 | 2.96 | 2.4 |
|  | Whitewater River (WHW) | Whitewater River to endorheic basin | 33.5694, -116.1080 | U, C | May 2015 | 2.75 | 1.6 |
| *^a^*F = Forests; R = Rangeland; D = Desert; U = Urban; C = Cropland  *^b^*Nearby croplands are largely irrigated pasture, on which insecticides are rarely used.  *^c^*No sediment was collected at Outlet Creek (OTL) as indicated by values of “--”. | | | | | | | |

**Table S1**. Sampling sites and dates of wild *H. azteca* collections in California including downstream waterbodies, latitude/longitude, the land uses in site vicinity, sediment organic carbon (OC), and chlorpyrifos concentrations in surficial sediments. nd =non-detected (<1 ng/g dry weight).

|  | Site Abbreviation | Screens*^a^*  (µm) | Body length  (mm; mean ± s.d.) | Control survival (%) | LC50  (ng/L (95% CI)) |
| --- | --- | --- | --- | --- | --- |
|  | | | | | |
| **Laboratory Animals** | | | | | |
|  | UCB laboratory | 500-600 | no data | 98 | 135 (115-158) |
|  |  | 500-600 | 2.4±0.3 | 93 | 172 (128-231) |
|  |  |  |  |  |  |
| **Low organophosphate and carbamate use (LowOCU) expected** | | | | | |
|  | LSH | 600-1000 | 3.3±0.6 | 93 | 181 (148-223) |
|  |  | 600-1000 | 3.3±0.6 | 90 | 219 (190-253) |
|  |  |  |  |  |  |
|  | OWN | 1000-2000 | 5.0±0.8 | 71 | 145 (108-194) |
|  |  |  |  |  |  |
|  | MJV | no data | 4.2±1.4 | 74 | 316 (233-430) |
|  |  | no data | 4.2±1.4 | 75 | 154 (116-205) |
|  |  |  |  |  |  |
| **Urban Sites** | | | | | |
|  | AMR | 500-600 | 2.3±0.3 | 78 | 132 (103-169) |
|  |  | 500-600 | 2.3±0.3 | 93 | 190 (161-225) |
|  |  |  |  |  |  |
|  | MED | 500-1000 | 3.3±0.7 | 95 | 676 (403-1130) |
|  |  |  |  |  |  |
|  | ESC | 500-1000 | 2.9±0.7 | 83 | 188 (125-282) |
|  |  |  |  |  |  |
| **Agricultural Sites (with or without urban influence)** | | | | | |
|  | ULC | 600-1000 | no data | 93 | 17,800 (9,220-34,200) |
|  |  |  |  |  |  |
|  | MSH | 500-600 | 2.1±0.2 | 95 | 542 (442-665) |
|  |  | 1000-2000 | 5.2±0.9 | 75 | 1120 (853-1470) |
|  |  |  |  |  |  |
|  | CHL | 1000-2000 | 4.9±0.7 | 88 | >1520*^b^* |
|  |  | 1000-2000 | 4.9±0.7 | 90 | >20,500*^b^* |
|  |  |  |  |  |  |
|  | CLG | 1000-2000 | 4.9±0.7 | 95 | >13,100*^c^* |
|  |  | 500-1000 | 3.2±0.6 | 78 | 156,000 (101,000-242,000) |
|  |  |  |  |  |  |

***^a^***Screen sizes are given as the size on which the animals were retained and the size through which they passed.

*^b­­^*There was 100% survival at the highest concentration tested of 1520 ng/L in the first test, and 67% survival in the second test at the highest concentration tested of 20,500 ng/L. The 20,500 ng/L value is a nominal concentration from the only test in the study for which an actual concentration was not available. Actual concentrations in all other tests throughout the study always fell between 69 and 120% of nominals.

*^c^*There was 67% survival at the highest concentration tested of 13,100 ng/L.

**Table S2**. Chlorpyrifos toxicity test (96-h) LC_50_ values from the University of California Berkeley (UCB) laboratory population and 10 field-collected populations of *H. azteca*. Data from multiple tests with the same population are shown when available (See **Table S1** for site abbreviations). No data are presented for OTL, BSC, BCM, KRN, RSN, BVS and WHW because the number of individuals available was insufficient for toxicity testing. CI = confidence intervals, s.d. = standard deviation.

| Primer/Primer Pair | | Left Primer (5’- 3’) | Right Primer (5’-3’) |
| --- | --- | --- | --- |
| *ace*-1 Amplification (cloning and genotyping assay) | | |  |
|  | I | TTCCGAAACCGAGACCTACC | TGACGTTGCAAGTGAAGTGG |
| *ace­*-1 Sequencing (cloning) | | |  |
|  | T7*^a^* | TAATACGACTCACTATAGGG |  |
|  | T3*^a^* |  | ATTAACCCTCACTAAAGGGA |
| *ace*-1 Sequencing (genotyping assay) | | |  |
|  | II |  | GATTGGGACAAACGGGAAGT |
|  | III |  | AACAAGTTACGGGAGAGGGG |
| COI Amplification | |  |  |
|  | IV*^b^* | GTTATATAAACTATTAGCCTTCCAA | ACTGCCACAACAGAYAARTAMGACCC |
|  | V*^b^* | GTTATATAAACTATTAGCCTTCCAA | ACAGCAACAACAGATAARTARGACC |
|  | VI*^b^* | GTTATATAAACTATTAGCCTTCCAA | ATCAAAATATACACYTCTGGGTGVCC |
| COI Sequencing | |  |  |
|  | VII*^b^* | GTTATATAAACTATTAGCCTTCCAA |  |
| *vgsc* Amplification | |  |  |
|  | VIII*^b,c^* | AGGGTGTTCAAGCTCGCTAA | ACATGCTCTCGATCCACTCC |
| *vgsc* Sequencing | |  |  |
|  | IX*^c^* |  | GGCCGTCTTGAGACCATTT |
|  | X*^c^* |  | ACATGCTCTCGATCCACTCC |
|  | XI*^b^* | AGGGTGTTCAAGCTCGCTAA |  |

| *^a^* Primers T7, T3 are from Invitrogen pCR®4-TOPO® TA Cloning® Kit (Invitrogen, Carlsbad, CA)  *^b^* Primers IV-VII, Lt Primer in pair VIII, primer XI and were designed by Weston et al. (2013).  *^c^* Rt primer in pair IX, primers X and XI were designed by Major et al. (2018).  **Table S3**. Primers used for *H. azteca* gene target cloning, amplification and/or sequencing. |
| --- |

|  |
| --- |

| **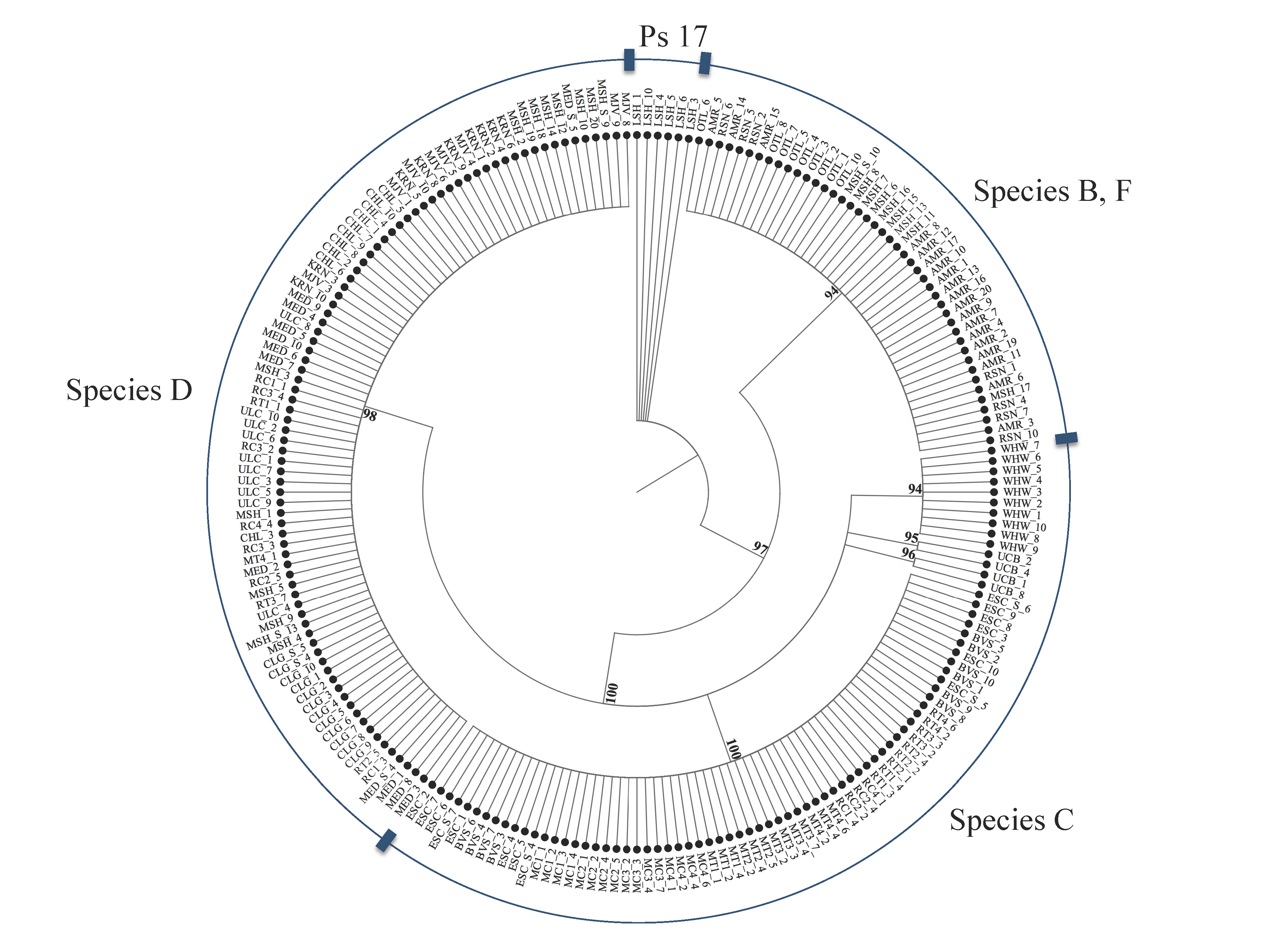**  **Fig S1**. Unrooted maximum likelihood (ML) cladogram of *H. azteca* based on a 326-bp segment (with 71 informative sites) of the voltage gated sodium channel (*vgsc*) for species determination of individuals for which acetylcholinesterase (*ace*-1) was genotyped. Terminal node labels represent individuals listed by site location (see **Table S1**) as well as the COI species group Bootstrap branch supports (10,000 replicates) above 90% are displayed. Outer circle labels represent species distinctions made by cytochrome *c* oxidase I (COI) analysis of a subset of *H. azteca* individuals within the *vgsc* cladogram. Based on the *vgsc* analysis, species C, D, and Ps 17 fall into distinct groups with strong support. Species B and F form a separate clade from other species groups, but are indistinguishable from one another based on the *vgsc* tree, while Species E and Ps 28 animals from the present study could not be included in the *vgsc* tree given that the *vgsc* assay was ineffective for this subset of animals. |
| --- |

| ULC Ind. |  | | | |  | | | |
| --- | --- | --- | --- | --- | --- | --- | --- | --- |
|  | M918 Codons | | M918 Amino Acids | | L925 Codons | | L925 Amino Acids | |
|  | 1 | 2 | 1 | 2 | 1 | 2 | 1 | 2 |
| 1 | ATG | TTG | M | L | CTC | ATC | L | I |
| 2 | ATG | ATG | M | M | ATC | ATC | I | I |
| 3 | ATG | ATG | M | M | ATC | ATC | I | I |
| 4 | ATG | TTG | M | L | CTC | ATC | L | I |
| 5 | ATG | ATG | M | M | ATC | ATC | I | I |
| 6 | ATG | ATG | M | M | ATC | ATC | I | I |
| 7 | ATG | TTG | M | L | CTC | ATC | L | I |
| 8 | ATG | ATG | M | M | ATC | GTC | I | V |
| 9 | ATG | ATG | M | M | ATC | ATC | I | I |
| 10 | ATG | ATG | M | M | ATC | ATC | I | I |

**Table S4**. Voltage gated sodium channel (*vgsc*) genotyping results showing pyrethroid resistance mutations at loci M918 and L925 from the 10 Ulatis Creek (ULC) wild individuals from the January 2018 collection. M918L, L925I, and L925V have been established as resistance mutations in *H. azteca*. All ULC individuals had at least one pyrethroid resistance allele. Frequencies of mutations were 0.15, 0.80, and 0.05 for M918L, L925I, and L925V, respectively.

|  |
| --- |
| **Fig S2**. An 876-bp alignment of the 20 different *H. azteca* acetylcholinesterase (*ace*-1) alleles obtained by cloning amplicons from a laboratory (UCB) and three wild populations (AMR, CLG, and MSH) (Part I). Survivors from 96-h chlorpyrifos toxicity tests were also included, and are represented by the populations labeled CLG_S and MSH_S. The individual from UCB is species C, while individuals from CLG/CLG_S and MSH/MSH_S are species D, and those from AMR are species B. Corresponding amino acid codes are listed above codons. Boxes denote sites with non-synonymous base pair substitutions relative to an allele from the laboratory population, and alternate amino acids are listed after the primary. |

|  |
| --- |
| **Fig S3**. An 876-bp alignment of the 20 different *H. azteca* acetylcholinesterase (*ace*-1) alleles obtained by cloning amplicons from a laboratory (UCB) and three wild populations (AMR, CLG, and MSH) (Part II). Survivors from 96-h chlorpyrifos toxicity tests were also included, and are represented by the populations labeled CLG_S and MSH_S. The individual from UCB is species C, while individuals from CLG/CLG_S and MSH/MSH_S are species D, and those from AMR are species B. Corresponding amino acid codes are listed above codons. Boxes denote sites with non-synonymous base pair substitutions relative to an allele from the laboratory population, and alternate amino acids are listed after the primary. |

|  |
| --- |
| **Fig S4**. An 876-bp alignment of the 20 different *H. azteca* acetylcholinesterase (*ace*-1) alleles obtained by cloning amplicons from a laboratory (UCB) and three wild populations (AMR, CLG, and MSH) (Part III). Survivors from 96-h chlorpyrifos toxicity tests were also included, and are represented by the populations labeled CLG_S and MSH_S. The individual from UCB is species C, while individuals from CLG/CLG_S and MSH/MSH_S are species D, and those from AMR are species B. Corresponding amino acid codes are listed above codons. Boxes denote sites with non-synonymous base pair substitutions relative to an allele from the laboratory population, and alternate amino acids are listed after the primary. An asterisk (*) indicates a stop codon. |

**Supporting Information Literature Cited**

Amweg, E. L. and D. P. Weston. 2007. Whole-sediment toxicity identification evaluation tools for pyrethroid insecticides: I. Piperonyl butoxide addition. *Environmental. Toxicololgy and Chemistry* 26 (11):2389-2396.

Major, Kaley M., Donald P. Weston, Michael J. Lydy, Gary A. Wellborn, and Helen C. Poynton. 2018. Unintentional exposure to terrestrial pesticides drives widespread and predictable evolution of resistance in freshwater crustaceans. *Evolutionary Applications*.

Weston, D. P., H. C. Poynton, G. A. Wellborn, M. J. Lydy, B. J. Blalock, M. S. Sepulveda, and J. K. Colbourne. 2013. Multiple origins of pyrethroid insecticide resistance across the species complex of a nontarget aquatic crustacean, *Hyalella azteca.* *Proceedings of the National Academy of Science* 110 (41):16532-7.
